# IMU-Based Joint Angle Measurement for Gait Analysis

**DOI:** 10.3390/s140406891

**Published:** 2014-04-16

**Authors:** Thomas Seel, Jorg Raisch, Thomas Schauer

**Affiliations:** 1 Control Systems Group (Fachgebiet Regelungssysteme), Technische Universitat Berlin, 10623 Berlin, Germany; E-Mails: raisch@control.tu-berlin.de (J.R.); schauer@control.tu-berlin.de (T.S.); 2 Systems and Control Theory Group, Max Planck Institute for Dynamics of Complex Technical Systems, 39106 Magdeburg, Germany

**Keywords:** inertial measurement units, gait analysis, gyroscopes and accelerometers, avoid magnetometers, exploit kinematic constraints, sensor-to-segment mounting, joint axis and position identification, joint angle measurement, validation against optical gait analysis, validation on prosthetic and human leg

## Abstract

This contribution is concerned with joint angle calculation based on inertial measurement data in the context of human motion analysis. Unlike most robotic devices, the human body lacks even surfaces and right angles. Therefore, we focus on methods that avoid assuming certain orientations in which the sensors are mounted with respect to the body segments. After a review of available methods that may cope with this challenge, we present a set of new methods for: (1) joint axis and position identification; and (2) flexion/extension joint angle measurement. In particular, we propose methods that use only gyroscopes and accelerometers and, therefore, do not rely on a homogeneous magnetic field. We provide results from gait trials of a transfemoral amputee in which we compare the inertial measurement unit (IMU)-based methods to an optical 3D motion capture system. Unlike most authors, we place the optical markers on anatomical landmarks instead of attaching them to the IMUs. Root mean square errors of the knee flexion/extension angles are found to be less than 1° on the prosthesis and about 3° on the human leg. For the plantar/dorsiflexion of the ankle, both deviations are about 1°.

## 1. Introduction

### 1.1. Inertial Measurement Units

Inertial sensors, also known as inertial measurement units (IMUs), measure acceleration, angular rate and the magnetic field vector in their own three-dimensional local coordinate system. With proper calibration, the axes of this local coordinate system represent an orthonormal base that is typically well aligned with the outer casing of the sensor. In addition to the mentioned inertial measurement signals, some commercially available devices incorporate algorithms that provide estimates of the sensor's orientation with respect to a global fixed coordinate system (see, e.g., [1]). This orientation can be represented by a quaternion, a rotation matrix or Euler angles. A number of algorithms have been proposed for sensor orientation estimation [2]. Typically, these algorithms employ strap-down-integration [3] of the angular rates to obtain a first estimate of the orientation. The drift in the inclination part of the IMU's orientation is eliminated using the assumption that the measured acceleration is dominated by gravitational acceleration [4]. Similarly, the estimation of the IMU's azimuth (or heading) requires the use of magnetometer measurements. Therefore, the presence of magnetic disturbances (as induced, e.g., by ferromagnetic material) may limit the accuracy of the orientation estimates, as demonstrated in [5,6]. We shall keep these limitations in mind, while we discuss methods for IMU-based joint angle estimation in the remainder of this article.

### 1.2. Robotic Hinge Joint vs. Human Knee

**Figure 1.**
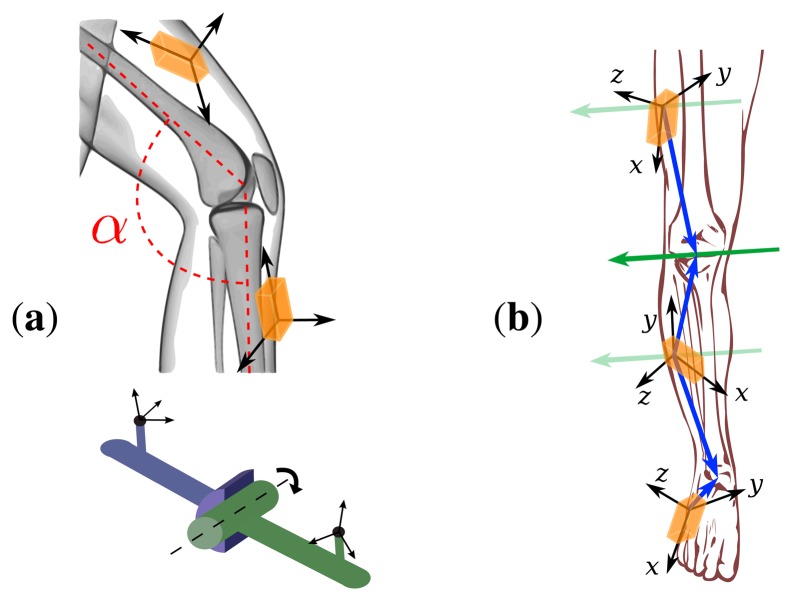
The placement of inertial sensors on the human body, the definition of joint angle and a model of a hinge joint. (**a**) The local sensor coordinate axes are not aligned with the physiological axes and planes by which the joint angle, *α*, is defined; (**b**) the coordinates of the joint axis direction (green arrows) and the joint position (blue arrows) in the local coordinate systems of the sensors characterize the sensor-to-segment mounting.

Since this contribution is concerned with IMU-based human gait analysis, we briefly highlight one of the major challenges of this task. Although many of the following statements are true in more general cases, we will focus our arguments on hinge joints (or pin joints, or knuckle joints), *i.e.*, joints with one rotational degree of freedom, as depicted in Figure 1. It has been demonstrated in many publications, e.g., [7] and the references therein, that inertial measurement data can be used to calculate hinge joint angles when at least one IMU is attached to each side of the joint. In most robotic and mechanical applications, the sensors can be mounted in such a way that one of the local coordinate axes coincides with the hinge joint axis; see, e.g., [7,8]. In that case, the hinge joint angle can be calculated by integrating the difference of both angular rates around the corresponding coordinate axis. Since even the most precise calibration will yield a non-zero bias, this calculated angle will be subject to drift. However, multiple techniques have been suggested to eliminate this effect using additional information from the accelerometers and/or the magnetometers, e.g., [7].

Similarly, inertial measurement units can be used to calculate hinge joint angles on the human body, for example on the knee joint (we will discuss the fact that the human knee is not a perfect hinge joint in Sections 2 and 3.3). However, there is a very important difference between the human leg and most robotic setups: It is very difficult to attach IMUs to the leg in such a way that one of the local coordinate axes coincides exactly with the knee joint axis. There have been some attempts (see, e.g., [9,10]), but since the human body lacks even surfaces and right angles, the accuracy of such approaches is limited. In contrast, the body straps that are commonly used to attach IMUs to the leg yield an almost arbitrary orientation of the IMU towards its segment, as illustrated in Figure 1. Nevertheless, the hinge joint angle can be calculated from the inertial measurement data. However, the data from both sensor units must be transformed into joint-related coordinate systems [11], *i.e.*, coordinate systems in which one or two axes coincide with the joint axis and/or the longitudinal axis of the segment. This is a major challenge in IMU-based joint angle measurement, not only on hinge-type joints. How it might be faced is discussed in Section 1.3 by reviewing common methods from the literature and in Section 3.1 by introducing new approaches that exploit the kinematic constraints of the joint. Furthermore, we will analyze in Section 2 how these techniques have been used by different authors to calculate knee joint angles. Finally, we will introduce a novel method for the measurement of flexion/extension angles on the knee and ankle in Section 3.2 and compare it to an established method in Section 4.

### 1.3. Arbitrary Mounting Orientation and Position

A fundamental problem in IMU-based human motion analysis is that the IMUs' local coordinate axes are not aligned with any physiologically meaningful axis; see Figure 1 for an illustration. First, we shall note that in some publications, this problem is ignored completely by assuming that the IMUs can be mounted precisely in a predefined orientation towards the joint; see, e.g., [9,10]. As can also be seen in the figures therein, this is a rather rough approximation. In the more realistic and, from a user's point of view, more convenient case of arbitrary mounting orientation, it is required to identify the joint axis coordinates in the local coordinate systems of the sensors attached to both ends of the joint.

**Figure 2.**
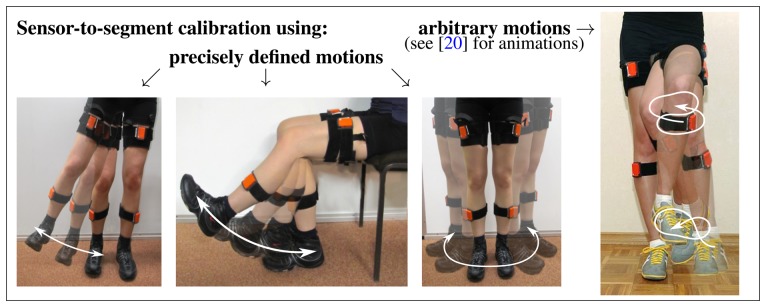
Examples for calibration motions that are used in the literature [14,15,17-19] to determine the coordinates of physiologically meaningful axes, e.g., the knee joint axis, in the local coordinate systems of the sensors. In such methods, the precision depends on how accurately the subject performs the motion. In contrast, the present approach uses arbitrary motions and identifies the sensor-to-segment mounting by exploiting kinematic constraints.

As illustrated in Figure 1, the sensor-to-segment mounting orientation and position are characterized by the local coordinates of the joint axis and the joint position, respectively. Both quantities might be measured manually, but in three-dimensional space, this is a cumbersome task that yields low accuracy results, as demonstrated, e.g., in [9,12]. Fortunately, at least for axis direction, alternatives exist. A common approach is to do this via calibration postures and/or calibration movements. Some authors, e.g., [13,14], make the subject stand with vertical, straight legs for a few seconds and use the acceleration measured during that time interval to determine the local coordinates of the segment's longitudinal axis. Additional sitting calibration postures are used in [13]. Besides static postures, predefined calibration motions can be used to identify the coordinates of physically meaningful axes in the upper and lower sensor coordinate system. Examples can be found in Figure 2 and in [14-16]. Moreover, a combination of postures and motions might be used to identify the sensor-to-segment orientations, as e.g., in the Outwalk protocol [17,18]. It employs pure flexion/extension motions and static poses to find the local coordinates of joint-related axes. Finally, the protocol used in [19] solves a closed kinematic chain to refine joint axis and position coordinates that have been obtained from a combination of calibration postures, predefined motion and manual measurements of body dimensions. However, it is important to note that, both in calibration postures and calibration motions, the accuracy is limited by the precision with which the subject can perform the postures or motions. Nevertheless, the mentioned methods for joint axis identification make a major contribution to the quality of IMU-based joint angle measurements. Therefore, most of the methods that are reviewed in Section 2 employ such techniques. In Section 3.1.1, we will introduce a new method that, unlike previous approaches, identifies the local joint axis coordinates from arbitrary motion data by exploiting kinematic constraints.

Besides the need of knowing the joint axis, some joint angle algorithms require additional knowledge of the joint position in local sensor coordinates; see, e.g., [9,21,22]. Furthermore, it has been demonstrated by Young [23] that joint position vectors can be used to improve the accuracy of body segment orientation estimates if the kinematic constraints of the joints are exploited. *Vice versa*, kinematic constraints have been used by Roetenberg *et al.* to estimate the joint positions based on accelerations and angular rates measured during motion, as briefly described in [21]. The method is also mentioned as an optional part of the body segment orientation Kalman filter described in [22]. In Section 3.1.3, we will propose a new method that exploits the same constraints, but uses a nonlinear least squares technique.

## 2. Brief Review of IMU-Based Knee Angle Estimation

Many algorithms and techniques have been suggested for IMU-based knee angle estimation. Despite the variety of approaches, the vast majority of authors defines the flexion/extension angle of the knee joint as the angle between the upper and lower leg along the main axis of relative motion, *i.e.*, the knee joint axis [9,13,14,24]. In other words, the projections of the upper and lower leg into the joint plane, to which the joint axis is normal, confine this angle; see Figure 1. However, we shall note that considering the knee as a hinge joint is an approximation. Although flexion/extension is the major degree of freedom, a biological joint, such as the knee, is not perfectly constrained to rotation around one axis. This is often addressed by additionally considering abduction/adduction and internal/external rotation, which leads to a three-dimensional knee joint angle, as in [10,14,25]. However, abduction/adduction and internal/external rotation angles hardly ever exceed a range of ±10° [14,26] and are strongly affected by soft-tissue artifacts [27,28]. Therefore, these additional degrees of freedom are not considered in many publications, e.g., [9,13,17,18,24].

As mentioned before, the simplest approaches in the literature assume that the IMUs are attached such that one of the local coordinate axes is aligned with the joint axis. Integrating the difference of the upper and lower sensor's angular rates around that axis will yield a drifting flexion/extension angle. In [10], this drift was removed using a high-pass filter. In another publication with the same mounting assumption, it was demonstrated that the joint angle can also be estimated from the measured accelerations if the position of the joint in both local coordinate systems is known [9]. Thereby, a root mean square error (RMSE) of less than 4° with respect to an optical reference system was achieved. Although both techniques may seem restricted to a special sensor mounting, they are just as helpful in the case of arbitrary mounting orientation, as long as the local joint axis coordinates are known.

A fundamentally different approach is found in [13]. After identifying the segment's longitudinal axis coordinates, the authors calculate the thigh's and shank's inclination and approximate the flexion/extension angle by the difference of these inclinations. Thereby, they achieve an RMSE of approximately 7° with respect to an optical reference system. However, their method is bound to the assumption that the knee axis remains horizontal during the entire motion. While that might be an acceptable approximation for most walking and running situations, this assumption does not hold during quick direction changes and for a number of other motions, like skating, hurdles or martial arts. In [24], the aforementioned method has been advanced. Instead of assuming a horizontal knee axis, the authors model the knee as a pure hinge joint and exploit its kinematic constraints using an extended Kalman filter. Thereby, they are able to calculate flexion/extension angles in good accordance with an optical reference system, both at the speed of running (8 km/h, RMSE < 4°) and walking (3 km/h, RMSE < 1°). Approximately the same precision for walking is achieved in [14]. Here, however, the complete orientation of each IMU with respect to a global reference coordinate system is calculated using a fusion algorithm that combines gyroscope and accelerometer measurements. Similarly, the algorithm used in [21,22] estimates sensor orientations from accelerations and angular rates. In [29], an RMSE below 4° was achieved by combining that algorithm with the Outwalk protocol mentioned in Section 1.3. Finally, a mean error (RMSE not available) below 2° was reported for the proprietary algorithm used in [30]. While it employs calibration poses and optional calibration motions to identify sensor-to-segment orientations (and, thus, the joint axis coordinates), the algorithm uses a biomechanical model and kinematic constraints to overcome integration drift [19].

It is important to note that almost all the mentioned RMS errors were obtained with the reference system markers being rigidly attached (usually in clusters on rectangular or L-shaped cardboard or plastic tiles) to the inertial sensors in order to eliminate the effect of soft tissue and skin motion artifacts on the measured joint angle difference [21,30]. The only exception from this statement is the work of Takeda *et al.* [13], who placed optical markers on anatomical landmarks, as it is common practice in optical gait analysis. However, they obtain a significantly larger RMS error than those authors who connected the reference markers to the IMUs. This means that most previous publications only compare the measurement accuracy of the optical and the inertial system, instead of comparing the results of an optical gait analysis to those of an inertial gait analysis. We believe that this aspect has received too little attention in previous publications. Therefore, we will place optical reference markers on anatomical landmarks during the experiments in Section 4, although this might increase the observed error.

Which of the reviewed methods is most suitable for a specific application depends also on the available sensor information. In many of the mentioned publications, the orientations of the thigh and shank are used to calculate the flexion/extension angle [13,14,18,24]. This is straight forward if reliable sensor orientation estimates are available and if the local joint axis coordinates are known. However, knowing the joint axis allows one to reduce the problem to one dimension immediately. Therefore, especially if reliable orientation estimates are not immediately available, it might be advantageous to use one of the methods in [9,10] instead or to combine them in a new way. We will examine both approaches in Sections 3.2 and 4.

## 3. New Methods for Inertial Sensor-Based Joint Angle Measurement

As explained in Section 1, handling arbitrary sensor-to-segment mounting is a major challenge in gait analysis with inertial sensors. Manual measurements, as well as calibration poses and movements, are commonly suggested solutions. Furthermore, we pointed out that the use of magnetometers is typically limited by the assumption of a homogeneous magnetic field. In this section, we describe a set of methods for IMU-based joint angle estimation that allow us to face these two challenges in a new way. We will combine elements of the methods reviewed above, but unlike most previous attempts, we will:

avoid sensor-to-segment mounting assumptions;
require no manual measurements of any distances, *etc.*;
not rely on the accuracy with which the subject performs predefined postures or movements;
and avoid the use of magnetometers.

Instead of employing any of these commonly used assumptions and restrictions, we make use of the fact that the knee joint behaves approximately like a mechanical hinge joint. The kinematic constraints that result from this fact are exploited to obtain the position vector and the direction vector of the knee flexion/extension axis in the local coordinates of both sensors. As outlined above, this information is crucial to precise joint angle calculation. We will use it to fill the gap between the sensor coordinate systems and the joint-related coordinate systems in which the angles are denned. Subsequently, this will allow us to calculate flexion/extension joint angles on joints with a major axis of motion, for example the knee and the ankle during walking. All of the methods that we will introduce use only angular rates and accelerations, while the use of magnetometer readings is completely avoided.

Before we describe the respective algorithms, let us define the available measurement signals. Assume that two inertial sensors, one attached to the upper leg and the other attached to the lower leg, measure the accelerations, *a*_1_(*t*),*a*_2_(*t*)ϵ ℝ^3^, and angular rates, *g*_1_(*t*),*g*_2_(*t*)ϵ ℝ^3^, at some sample period, Δ*t*. Additionally, we calculate the time derivatives *ġ*_1_(*t*),*ġ*_2_(*t*)ϵ ℝ^3^ of the angular rates via the third order approximation:

(1) $${\dot{g}}_{1/2}(t)\approx \frac{g_{1/2}(t-2\Delta t)-8g_{1/2}(t-\Delta t)+8g_{1/2}(t+\Delta t)-g_{1/2}(t+2\Delta t)}{12\Delta t}$$

### 3.1. Identification of the Joint Axis and Position

Both the location of the sensors on the segments and their orientation with respect to the segments are assumed to be completely unknown. In particular, we do not assume that any of the local sensor axes coincides with the knee joint axis or the longitudinal axis of the segment or bone. Therefore, neither the direction nor position of the knee flexion/extension axis are known. However, these coordinates can be identified from the measurement data of arbitrary motions by exploiting kinematic constraints, as explained in [12]. The first step of this identification is the gathering of identification data, while the knee is moved around its degrees of freedom in an arbitrary manner (*i.e.*, we do *not* assume any type of particular motion, like walking or motions in a certain direction). About every tenth of a second, a dataset, *S*(*i*), of the form:

(2) $$S(i)=\{a_{1}(t_{i}),a_{2}(t_{i}),g_{1}(t_{i}),g_{2}(t_{i}),{\dot{g}}_{1}(t_{i}),{\dot{g}}_{2}(t_{i})\}$$

is recorded (of course, the time between taking two datasets must be a multiple of the sample period, *i.e.*, Δ*t*|(*t_i_*_+1_ — *t_i_*) ∀*i*). Thereby, a total number of *N* ≫ 1 datasets are collected, which will be used in the subsequent sections to identify local joint axis and position coordinates.

#### 3.1.1. Identification of the Joint Axis Coordinates

The datasets, *S*(*i*), *i* ϵ [1, *N*], are used to identify the unit-length direction vectors, *j*_1_, *j*_2_ ϵ ℝ^3^, of the knee flexion/extension axis in the local coordinates of both sensors. It is important to note that *j*_1_ and *j*_2_ are constants and depend only on the orientation in which the sensor is mounted with respect to the joint. As explained in [12], the angular rates, *g*_1_(*t*), *g*_2_(*t*), measured on a hinge joint differ only by the joint angle velocity vector and a (time-variant) rotation matrix. Hence, their projections into the joint plane (*i.e.*, the geometrical plane to which the joint axis is the normal vector) have the same lengths for each instant in time, which is equivalent to:

(3) $${\left\|g_{1}(t)\times j_{1}\right\|}_{2}-{\left\|g_{2}(t)\times j_{2}\right\|}_{2}=0\forall t$$

where ‖ · ‖_2_ denotes the Euclidean norm. This constraint holds regardless of where and in which orientation the sensors are mounted on the segments. In particular, every dataset, *S*(*i*), *i* ϵ [1, *N*], must fulfill Equation (3). We can therefore identify *j*_1_ and *j*_2_ by minimizing the left-hand side of Equation (3) for all datasets in a least squares sense. More precisely, we write *j*_1_ and *j*_2_ in spherical coordinates:

(4) $$j_{1}={\left(\cos(\phi_{1})\cos(\theta_{1}),\cos(\phi_{1})\sin(\theta_{1}),\sin(\phi_{1})\right)}^{T}$$

(5) $$j_{2}={\left(\cos(\phi_{2})\cos(\theta_{2}),\cos(\phi_{2})\sin(\theta_{2}),\sin(\phi_{2})\right)}^{T}$$

and define the sum of squared errors:

(6) $$\begin{array}{cc} \Psi (\phi_{1},\phi_{2},\theta_{1},\theta_{2}):=\sum_{i=1}^{N}e_{i}^{2}, & e_{i}={\left\|g_{1}(t_{i})\times j_{1}\right\|}_{2}-{\left\|g_{2}(t_{i})\times j_{2}\right\|}_{2}(6) \end{array}$$

**Figure 3.**
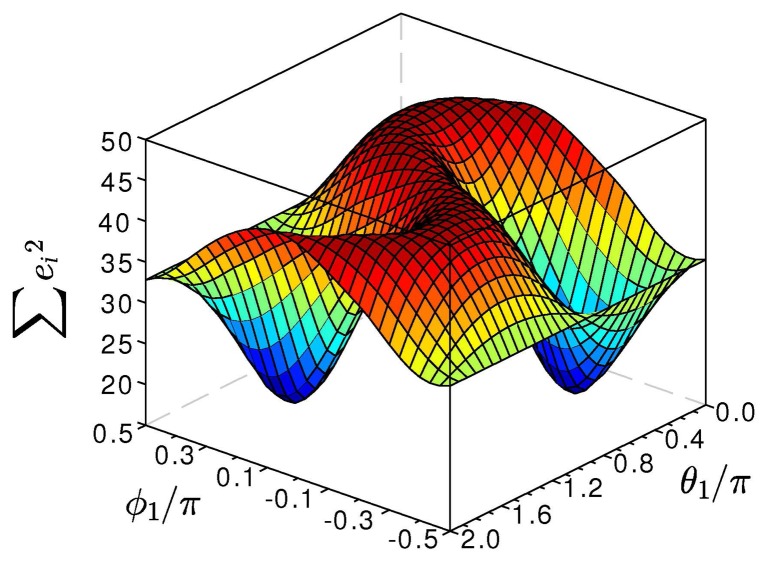
Sum of squares ψ(*j*_1_, *j*_2_) of the error in the kinematic constraint (3). The two minima represent the true local coordinates, *j*_1_ and — *j*_1_, of the joint axis direction vector.

Figure 3 depicts the typical form of this cost function. Since Equation (3) is invariant with respect to the signs of *j*_1_ and *j*_2_, this cost function has four minima, which correspond to the four possible combinations of signs, (*j*_1_, *j*_2_), (-*j*_1_, *j*_2_), (*j*_1_, -*j*_2_) and (-*j*_1,_, *-j*_2_). By minimizing ψ(*ϕ*_1_, *ϕ*_2_, *θ*_1_, *θ*_2_) over its arguments, we identify these true joint axis coordinates. This optimization might be implemented using a Gauss-Newton algorithm, as further described in [12], or any other standard optimization method [31].

#### 3.1.2. Matching Signs of the Joint Axis Coordinates

**Figure 4.**
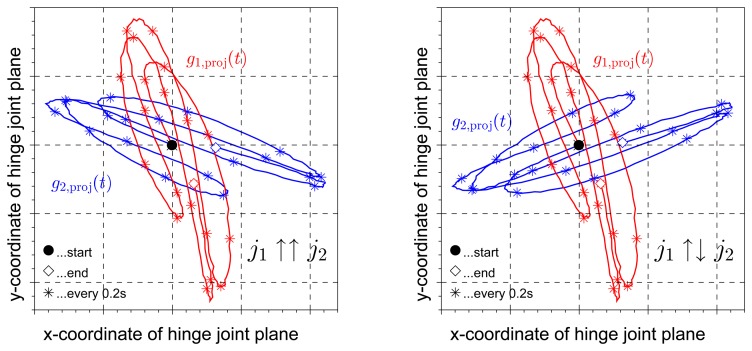
Projection of the measured angular rates of both sensors into the joint plane (defined by the coordinates in Equation (13)) for a motion with little flexion/extension. In both plots, the projections have the same length at each moment in time, *cf*. Equation (3). However, when the joint axis signs match, the two curves are congruent up to some rotation around the origin, while in the case of opposite signs, they are mirror images of each other.

In Section 3.2, we will use *j*_1_ and *j*_2_ to approximate the gyroscope-based joint angle velocity by *g*_1_(*t*) · *j*_1_- *g*_2_(*t*) · *j*_2_. Therefore, it is important to ensure that the signs of *j*_1_ and *j*_2_ match, *i.e.*, that they point to the same direction. In practice, this can easily be achieved by a quick look at the sensor's mounting orientation. An example is given in Figure 1b, where the z-axis of both sensors point roughly laterally (*i.e.*, the coordinate axis points into the lateral half space, which is an easy observation; we do not restrict the mounting orientation in any way). If instead, to give another example, the local *y*-axis of the first sensor points roughly medially, while the local *z*-axis of the second sensor points roughly laterally, then the *x*-coordinate of *j*_1_ and the *z*-coordinate of *j*_2_ should have opposite signs. In case the mounting of the sensors cannot be observed, the correct pairing of the signs can also be determined from the inertial data itself. As a first step, we choose a period from the identification data during which the angular velocities around the joint axis were negligible, *i.e.*, *g*_1_(*t*) · *j*_1_ ≈ 0, *g*_2_*(t)* · *j*_2_ ≈ 0. Then, as demonstrated in Figure 4, the traces of the angular rates in the local joint planes of the two sensors reveal the correct pairing. They are congruent up to rotation if the signs match, and they are rotated mirror images of each other if the signs do not match. For the present data analysis, this step is implemented as an automatic routine in the joint axis identification algorithm.

#### 3.1.3. Identification of the Joint Position Coordinates

For a number of methods in the literature and for one of the methods that will be introduced in Section 3.2, it is useful to determine the position of the sensors with respect to the joint, *i.e.*, in other words, the joint center position in the local coordinates of the sensors. Again, it should be noted that the vectors, *o*_1_, *o*_2_ ϵ ℝ^3^, from the joint center to the origin of the first and the second sensor frame are constants that do not change during motion and only depend on the mounting position and orientation.

A method is introduced in [12] that allows us to determine these quantities on spheroidal joints from the inertial data of arbitrary motions that excite all degrees of freedom of the joint. It exploits the fact that the acceleration of each sensor can be thought of as the sum of the joint center's acceleration and the acceleration due to the rotation of that sensor around the joint center. Apparently, the acceleration of the joint center must be the same in both local frames, up to some time-variant rotation matrix that corresponds to the rotation of both local frames to each other. Mathematically, this constraint is expressed by:

(7) $$\begin{array}{c} {\left\|a_{1}(t)-\Gamma_{g_{1}(t)}(o_{1})\right\|}_{2}-{\left\|a_{2}(t)-\Gamma_{g_{2}(t)}(o_{2})\right\|}_{2}=0\forall t\\ \Gamma_{g_{i}(t)}(o_{i}):=g_{i}(t)\times (g_{i}(t)\times o_{i})+{\dot{g}}_{i}(t)\times o_{i},i=1,2 \end{array}$$

where Γ*_g_i__*_(_*_t_*_)_(*o_i_*) is the radial and tangential acceleration due to rotation around the joint center. By subtracting Γ*_g_i__*_(_*_t_*_)_(*o_i_*), the measured acceleration, *a_i_*(*t*), is shifted by — *o_i_* yielding the acceleration of the joint center.

In [12], this argument is given for spheroidal joints only. However, the very same constraint also holds on a hinge joint. Every point on the hinge joint axis is a solution of Equation (7). More precisely, every pair of coordinates, *o*_1_, *o*_2_, that describes a point on the joint axis fulfills that constraint for any given motion that the joint might perform. Therefore, we use the same arbitrary motion data, *S*(*i*), *i* ϵ [1, *N*], as in Section 3.1.1, and define another sum of squared errors:

(8) $$\tilde{\text{Ψ}}(o_{1},o_{2}):=\sum_{i=1}^{N}e_{1}^{2},\;e_{i}={\left\|a_{1}(t)-\Gamma_{g_{1}(t)}(o_{1})\right\|}_{2}-{\left\|a_{2}(t)-\Gamma_{g_{2}(t)}(o_{2})\right\|}_{2}$$

We minimize ψ̃ (*o*_1_,*o*_2_) over its arguments via a Gauss-Newton algorithm, the implementation of which is described in [12]. As mentioned above, any other optimization method [31] might be employed as well. Since the result of that optimization, denoted by *ô*_1_, *ô*_2_, refers to an arbitrary point along the joint axis, we shift it as close as possible to the sensors by applying:

(9) $$\begin{array}{cc} o_{1}={\hat{o}}_{1}-j_{1}\frac{{\hat{o}}_{1}\cdot j_{1}+{\hat{o}}_{2}\cdot j_{2}}{2}, & o_{2}={\hat{o}}_{2}-j_{2}\frac{{\hat{o}}_{1}\cdot j_{1}+{\hat{o}}_{2}\cdot j_{2}}{2} \end{array}$$

which uses the previously identified joint axis coordinates. For the present data analysis, this step is implemented as the final step of an automatic algorithm for joint position identification.

### 3.2. Calculation of the Flexion/Extension Angle

We assume that the local joint axis coordinates, *j*_1_, *j*_2_, and the local joint position coordinates, —*o*_1_,—*o*_2_, have been successfully identified using the methods described above. As explained in Section 1.3, this is crucial for IMU-based joint angle measurement. The identified values of *j*_1_, *j*_2_ and *o*_1_, *o*_2_ are now used to calculate the flexion/extension angle of an anatomical joint with one major degree of freedom. While we consider a knee joint to explain the methods, we extend them to the more general case of saddle and spheroidal joints in Section 3.3.

**Figure 5.**
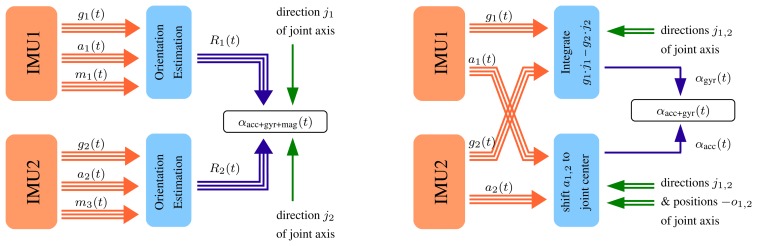
Two algorithms for IMU-based knee angle calculation are considered. **(Left)** Sensor orientation estimates are used to calculate the orientational difference (*i.e.*, the joint angle) around a given axis. **(Right)** The problem is reduced to one dimension immediately by integrating the difference of the angular rates around the joint axis. Then, an acceleration-based joint angle estimate is used to remove drift.

Figure 5 shows the main ideas of the two methods for joint angle measurement that we will describe. The first method assumes that each IMU provides highly accurate estimates of its orientation with respect to a common fixed reference coordinate system. Together with the local joint axis coordinates, these orientations directly yield an accurate flexion/extension angle. This approach is well known from the literature [13,14,29]. The second and novel method reduces the problem to the joint plane from the very start by integrating both angular rates only around the joint axis, which yields a highly accurate, but slowly drifting, joint angle. This angle is combined in a sensor fusion with a noisy, but driftless, joint angle estimate that is calculated from the measured accelerations. At this point, the second method also uses the joint position vectors, but unlike the first method, it does not rely on magnetometer readings.

#### 3.2.1. Joint Angle from Sensor Orientation Estimates

As mentioned previously, some inertial sensors include on-board orientation estimation, which is usually based on a sensor fusion of the acceleration, angular rate and magnetic field vector measurements. These estimates describe the orientation of the sensors with respect to a fixed reference coordinate system, either in quaternions, rotation matrices or Euler angles. As mentioned in Section 2, it is an established method to use sensor orientation estimates for the calculation of joint angles; see, e.g., [13,14,29]. In the following, we assume that the orientation of both sensors with respect to a common fixed reference frame (*i.e.*, the reference frame must be identical for each sensor) are given by rotation matrices, which we denote by *R*_1_(*t*) and *R*_2_(*t*). They shall be defined, such that they transform a locally measured vector into the reference frame, *i.e.*, we have *R*_1_(*t*)*j*_1_ = *R*_2_(*t*)*j*_2_∀*t*. Under these circumstances, the flexion/extension angle *α*_acc+gyr+mag_(*t*) can simply be computed as:

(10) $$\begin{array}{cc} \alpha_{\text{acc}+\text{gyr}+\text{mag}}(t)=;{∢}_{3d}(R_{1}(t)(j_{1}\times c),(R_{2}(t)(j_{2}\times c)), & c∦j_{1},c∦j_{2} \end{array}$$

where ∢_3_*_d_*() denotes the (signed) angle between two vectors in ℝ^3^ and *c* ϵ ℝ^3^ can be any vector that makes none of the vector products zero (e.g., *c* = [1,0,0]*^T^* can be used, unless *j*_1_ or *j*_2_ happens to be exactly [±1,0, 0]*^T^*). It is important to note that, by construction, this joint angle can only be as precise as the employed sensor orientation estimates, and it might be drifting if the orientation estimates are drifting.

#### 3.2.2. Joint Angle from Accelerometer and Gyroscope Readings

In the following, we will compute the flexion/extension angle only from accelerations and angular rates. A gyroscope-based flexion/extension angle can be calculated by integrating the difference of the angular rates around the joint axis, *i.e.*,

(11) $$\alpha_{\text{gyr}}(t)=\int_{0}^{t}(g_{1}(\tau )\cdot j_{1}-g_{2}(\tau )\cdot j_{2})d\tau$$

Furthermore, the knowledge of the joint axis coordinates allows us to employ many of the restrictive methods from the literature reviewed above, which require the sensor axes to coincide with joint axes or segment axes. In particular, we can extend the approach used in [9] to three-dimensional space. We shift the measured accelerations onto the joint axis by applying:

(12) $$\begin{array}{cc} {\tilde{a}}_{1}(t)=a_{1}(t)-\Gamma_{g1(t)}(o_{1}), & {\tilde{a}}_{2}(t)=a_{2}(t)-\Gamma_{g2(t)}(o_{2}) \end{array}$$

with Γ*_g_*_1/2_(*t*)(*o*_1/2_) defined in Equation (7). As explained in Section 3.1.3, *ã*_1_(*t*) and *ã*_2_(*t*) are the same quantity measured in two different local coordinate systems, which rotate with respect to each other around one axis. Therefore, the flexion/extension angle can be approximated by the angle between the projections of *ã*_1_(*t*) and *ã*_2_(*t*) into the joint plane (ideally, the two angles are identical, but due to measurement inaccuracies, it is rather an approximation). Consequently, we define a pair of joint plane axes *x*_1/2_, *y*_1/2_ ϵ ℝ^3^ for each local frame:

(13) $$\begin{array}{ccccc} x_{1}=j_{1}\times c, & y_{1}=j_{1}\times x_{1}, & x_{2}=j_{2}\times c, & y_{2}=j_{2}\times x_{2}, & c∦j_{1}, \end{array}c∦j_{2},$$

and we calculate the accelerometer-based joint angle by:

(14) $$\alpha_{\text{acc}}(t)={∢}_{2d}\left(\left[\begin{array}{c} {\tilde{a}}_{1}(t)\cdot x_{1}\\ {\tilde{a}}_{1}(t)\cdot y_{1} \end{array}\right],\left[\begin{array}{c} {\tilde{a}}_{2}(t)\cdot x_{2}\\ {\tilde{a}}_{2}(t)\cdot y_{2} \end{array}\right]\right)$$

where∢_2_*_d_*() denotes the (signed) angle between two vectors in ℝ^2^. The resulting angle, *α_acc_*(*t*), is not affected by drift, since we did not employ any integration to calculate it. We shall note that the above equations are sensitive to measurement errors if the shifted accelerations, *ã*_1/2_(*t*), are almost collinear with the joint axes *j*_1/2_. However, in almost every practical situation, the gravitational acceleration dominates the acceleration signals *a*_1_(*t*), *a*_2_(*t*) and *ã*_1_(*t*), *ã*_2_(*t*). Therefore, the errors should only be significant when the knee axis is close to vertical or during the periods in which the knee is strongly accelerated in the medial or lateral direction. Both situations are rare in walking and most other motions of sports or daily activities. Please also note that Γ_g1/2_(*t*)(*o*_1/2_) in Equation (12) is typically small compared to gravitational acceleration and therefore sensitivity to inaccuracies in *o*_1_, *o*_2_ is low.

**Figure 6.**
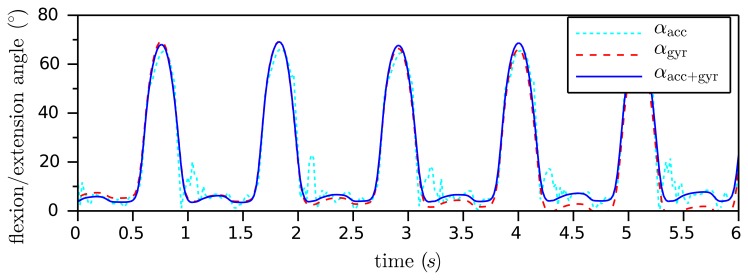
Sensor fusion of the gyroscope-based and the accelerometer-based knee angle of a leg prosthesis. The noisy, but driftless, angle, *α*_acc_(*t*), is combined with the very precise, but drifting, angle, *α*_gyr_(*t*), using the complementary filter (15). The resulting angle, *α*_acc+gyr_(*t*), is accurate on small and on large time scales.

Figure 6 shows the typical course of the two angles, *α*_gyr_(*t*) and *α_acc_*(*t*), that we derived in this subsection. The gyroscope-based angle is very precise on short time scales, but exhibits some slow drift of about 1.5 °/s (please note that the drift depends on the the bias of the gyroscopes). The accelerometer-based angle does not drift, but it is affected by the accelerometer noise and seems to be less reliable in moments of large acceleration changes. Therefore, it is advantageous to combine both angles using a standard tool of sensor fusion, e.g., a complementary filter [32] or a Kalman filter. The result shall be denoted by *α*_acc+gyr_(*t*). A simple implementation example is given by:

(15) $$\begin{array}{cc} \alpha_{\text{acc}+\text{gyr}}(t)=\lambda \alpha_{\text{acc}}(t)+(1-\lambda )(\alpha_{\text{acc}+\text{gyr}}(t-\Delta t)+\alpha_{\text{gyr}}(t)-\alpha_{\text{gyr}}(t-\Delta t)), & \lambda \in [0,1] \end{array}$$

Figure 6 presents the result of the sensor fusion for a weight λ = 0.01 and a sample period Δ*t* = 0.02 s. As demonstrated, *α*_acc+gyr_(*t*) does not follow the spikes of the acceleration-based angle and also does not exhibit the drift of the gyroscope-based angle. In Section 4, we will examine how accurate this IMU-based flexion/extension angle measurement is.

### 3.3. Extension to Saddle and Spheroidal Joints

The method that was introduced in the previous subsection assumes that two segments are connected by a joint with one rotational degree of freedom. As mentioned before, the human knee is not exactly such a hinge joint, since it admits some rotation in the frontal and the transversal plane of up to about 8° [26]. These motions are even stronger when saddle or spheroidal joints, e.g., the ankle or the hip, are considered. Therefore, we briefly discuss the influence of these additional motions on the methods proposed above. Since the joint position estimation introduced in Section 3.1.3 exploits the kinematic constraint of a spheroidal joint, it works just as well when such motions occur, as demonstrated in [12] for ankle joints. Likewise, the joint axis estimation, which exploits the kinematic constraint (3) of a hinge joint, can be employed on saddle and spheroidal joints. However, it will always identify the main axis of motion, *i.e.*, the axis that minimizes the sum of squares in (6). This means that other motion may occur. However, while the identification data is recorded, flexion/extension must be dominant in order to obtain the corresponding axis. In Section 4, we will demonstrate that, in the case of the ankle joint, data from normal walking is sufficient to properly identify the dorsiflexion/plantarflexion axis.

The calculation of joint angles, as described above, is limited to rotations around the identified joint axis in both methods. While both methods might also be adapted and employed for abduction/adduction and inversion/eversion angle measurements, we focus only on flexion/extension. As mentioned above, this is in accordance with numerous authors [9,13,17,18,24]. Nevertheless, small additional rotations in the other dimensions do not affect any of the geometrical arguments used in the algorithms above. Therefore, they can be employed for flexion/extension angle measurement on real saddle or spheroidal joints, e.g., the hip or the ankle. In Section 4, we will examine how accurate these methods work on the plantar/dorsiflexion of ankle joints.

## 4. Experimental Results and Discussion

**Figure 7.**
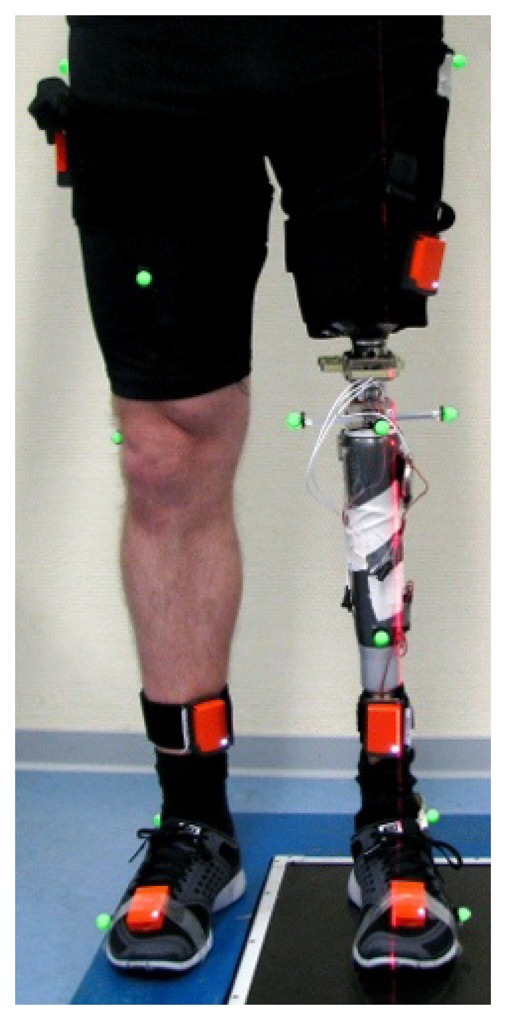
Placement of inertial measurement units and optical markers on the legs of a transfemoral amputee. The optical markers are placed at the typical physiological landmarks. The IMUs are attached using body straps without restricting their position or orientation.

The two methods that were introduced in Section 3 are now evaluated in repeated gait experiments with a transfemoral amputee (age 40, height 182 cm, weight 83 kg, K-Level 4, *i.e.*, the highest level of the Amputee Mobility Predictor). The subject is wearing a leg prosthesis and has given informed consent to the investigations. Reflection markers are placed on the body segments of the subject at corresponding physiological landmarks; see Figure 7. The 3D positions of these markers are recorded at 120 Hz by an optical motion tracking system with ten cameras (Vicon V612 [33]). Furthermore, we use elastic body straps to equip the upper and lower leg, as well as the foot, of both the prosthesis and the contralateral leg with one inertial measurement unit (Xsens MTw [1]) each, as depicted in Figure 7. At a measurement rate of 60 Hz, these six devices provide 3D accelerations and angular rates in local coordinates, as well as estimates of sensor orientations with respect to a common global reference frame. We neither restrict the mounting of the IMUs to certain locations or orientations, nor do we measure these quantities. Instead, the subject is instructed to perform circling motions of the upper leg, the lower leg and the foot with a few arbitrary changes in direction and amplitude. This motion is executed for about ten seconds on both sides (see Figure 2 and [20] for an illustration). The methods from Sections 3.1.1 and 3.1.3 are used to estimate the knee axis direction and position, as well as the ankle joint position on both sides from the recorded inertial data. Subsequently, the subject walks repeatedly about ten meters at a self-selected speed on a straight line within the range of an optical gait analysis system and far away from potential magnetic disturbances. The data that is gathered during these walking trials is, on the one hand, used to identify the ankle plantar/dorsiflexion axis on both sides. On the other hand, we calculate the knee flexion/extension angles and the ankle plantar/dorsiflexion angle of both legs using the methods from Sections 3.2.1 and 3.2.2.

**Figure 8.**
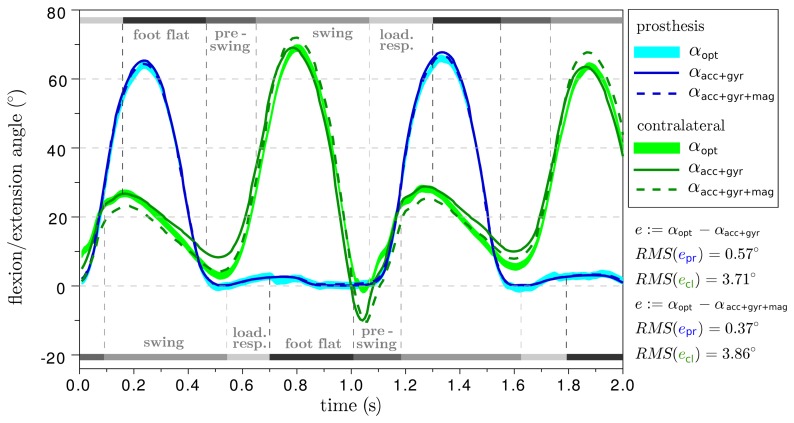
Comparison of the two IMU-based knee flexion/extension angle measurements (*α*_acc+gyr+mag_(*t*) and *α*_acc+gyr_(*t*)) with the result of an optical gait analysis system (*α*_opt_(*t*)). On the prosthesis side, there is no significant deviation (*e*_pr_ < 0.6°). However, on the contralateral side, skin and muscle motion effects, which are strongest during push-off and heel-strike, lead to RMS errors *e*_cl_ of almost 4°.

**Figure 9.**
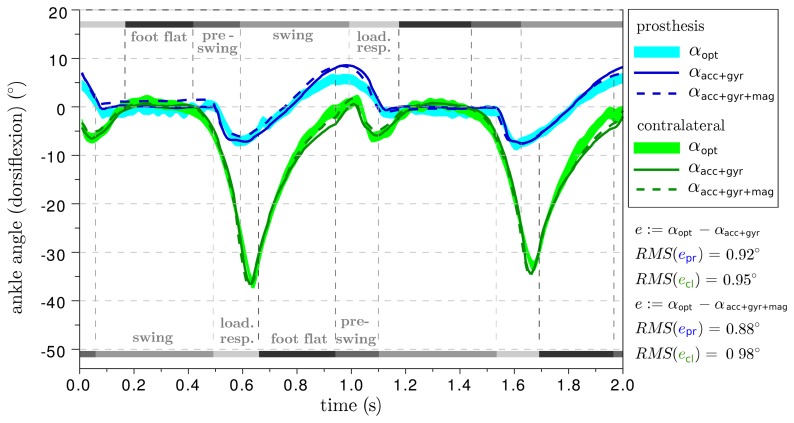
Comparison of the two IMU-based ankle plantar/dorsiflexion angle measurements (*α*_acc+gyr+mag_(*t*) and *α*_acc+gyr_(*t*)) with the result (*α*_opt_(*t*)) of an optical gait analysis system. Both on the prosthesis side and on the contralateral side, the deviation is about 1°.

The resulting ankle and knee angle traces of two different trials are provided in Figures 8 and 9. The difference between the prosthesis and the human leg is considerable, but this aspect is outside the focus of this manuscript and, therefore, shall not be discussed here. For additional orientation, gait phase transitions are indicated, which were detected based on inertial measurement data from the foot sensor using an offline version of the algorithm described in [34,35]. With respect to the optical system, both IMU-based methods achieve a root-mean-square deviation of less than 0.6° on the prosthesis side and more than 3° on the contralateral side. The deviations of the subsequent trials are summarized in Table 1. In all trials, both IMU-based approaches yield similar values, although they use the inertial data in completely different ways.

It is important to note that the errors on the human leg are about four times larger than on the prosthesis. One might suppose that this is because of the human knee being less close to a perfect hinge joint. However, we just explained in Section 3.3 that the IMU-based algorithms ignore abduction/adduction and internal/external rotations, just as the optical analysis does. Therefore, a more reasonable explanation is found in the following remarkable difference between the two sides: on the prosthesis, the IMUs and the optical markers are rigidly connected by the artificial thigh and shank. However, on the human leg, the inertial sensors and the markers move relative to each other as a result of muscle and skin motions. This argument is supported by the fact that deviations between optical and IMU-based angles are largest during push-off and heel strike, *i.e.*, when the leg is accelerated and decelerated. Furthermore, there are a number of experimental studies (see Section 2) in which the optical markers were placed directly on the inertial sensors or onto rigid plastic or wood parts that also held the inertial sensors. In these studies, deviations of less than 2° were observed, which further supports the argument that the deviations in Figure 8 result from skin and muscle motions.

## 5. Conclusions

In the previous sections, we discussed methods for IMU-based joint angle measurement on the human body. Special attention was dedicated to the challenge of arbitrary mounting orientation and position. We proposed a set of methods that allow us to determine the local joint axis and position coordinates from arbitrary motions by exploitation of the kinematic constraints of the joint. We believe that these methods are more practical and more robust than previously suggested methods that require the subject to perform a precise calibration movement or pose than methods that require one to attach the sensors in specific positions or orientations

In addition, we described two methods for the calculation of precise flexion/extension angles on hinge, saddle and spheroidal joints. The first method is known from the literature and requires precise estimates of the sensors' orientations with respect to a common fixed reference frame. The second and novel method employs only accelerometer and gyroscope readings. Since the use of magnetometers is avoided, it can be used indoors and in the proximity of magnetic disturbances. Both methods were evaluated against an optical gait analysis system on the gait of a transfemoral amputee. We obtained highly precise results with RMSE of about 1° on the ankle joints, as well as on the prosthetic knee, and discussed the effect of skin and muscle motions on the contralateral knee, which led to slightly larger deviations of about 3°. Future research will be dedicated to the question of how these effects can be compensated for or minimized.

Furthermore, the proposed algorithms are such that it is straight forward to implement them for online use. Therefore, and since they supersede manual measurements and precise calibration movements, these new methods open the door to a plug-and-play gait analysis, in which one simply attaches the IMUs, moves the legs for a few seconds and then receives joint angle measurements in real time. This will be the subject of our future research, including extensions for 2D and 3D angle measurements on ankle and hip joints.

## Figures and Tables

**Table 1.** Deviations between the knee flexion/extension and ankle plantar/dorsiflexion angle measurements of the optical and the inertial system for six gait trials of a transfemoral amputee. Results are given for *α*_acc+gyr_ only, since both methods yield very similar results. The deviations vary little (*σ* ≈ 1°). For the knee angles, the same difference in the accuracies of the prosthesis and human leg is observed as in Figure 8.

		**Trials**						**RMSE**	
		1	2	3	4	5	6	*σ*	**Average**
**knee**	prosthesis	0.46°	0.89°	0.59°	0.95°	0.57°	0.77°	0.19°	**0.71**°
	contralateral	3.25°	2.76°	3.10°	3.16°	0.40°	3.83°	1.20°	**3.30**°
**ankle**	prosthesis	0.92°	1.03°	0.91°	0.65°	0.67°	0.69°	0.16°	**0.81**°
	contralateral	0.95°	1.50°	1.25°	1.53°	1.85°	2.61°	0.57°	**1.62**°

## Notes

**Author Contributions:** Thomas Seel developed and implemented the algorithms for joint axis and position identification and for joint angle measurement under the valuable guidance and advice of Thomas Schauer. Both prepared the data acquisition for the experiments that the acknowledged colleagues and Thomas Seel carried out. Jorg Raisch is the head of the research group in which the reported research was performed. His contribution to the research was general guidance and advice. All authors contributed in writing and revising the manuscript.
